# Applications in Awake Animal Magnetic Resonance Imaging

**DOI:** 10.3389/fnins.2022.854377

**Published:** 2022-04-05

**Authors:** Craig F. Ferris

**Affiliations:** Center for Translational NeuroImaging, Northeastern University, Boston, MA, United States

**Keywords:** fear, drug abuse, pain, epilepsy, phMRI (pharmacological fMRI)

## Abstract

There are numerous publications on methods and applications for awake functional MRI across different species, e.g., voles, rabbits, cats, dogs, and rhesus macaques. Each of these species, most obviously rhesus monkey, have general or unique attributes that provide a better understanding of the human condition. However, much of the work today is done on rodents. The growing number of small bore (≤30 cm) high field systems 7T- 11.7T favor the use of small animals. To that point, this review is primarily focused on rodents and their many applications in awake function MRI. Applications include, pharmacological MRI, drugs of abuse, sensory evoked stimuli, brain disorders, pain, social behavior, and fear.

## Introduction

Between 1998 and 2000 there were three studies on awake imaging in rhesus monkeys (Dubowitz et al., 1998; Stefanacci et al., 1998; Zhang et al., 2000). These were all done in large bore human 1.5T scanners with surgical implants in the skull used to immobilize the head. My lab published the first paper on awake rat imaging in 1999 (Lahti et al., 1999). The system was a General Electric CSI-II 2.0T small bore scanner for animal research. In the early 1990s GE was essentially giving these small scanners away with any purchase of their large bore, clinical 1.5T Signa scanner to encourage researchers to develop new pulse sequences and protocols that could eventually migrate to the console of the human scanner. Coming from a Psychiatry Department, I reasoned this new field of non-invasive MRI could help understand the etiology and pathophysiology of mental illness. A normal brain with some genetic risk could be subjected to an environmental insult early in life and followed into adulthood and the onset of mental illness. Mental health professionals use the phrase “diathesis and stress” to describe this cause and effect. This type of prospective, longitudinal study inflicting early insult and following brain function with aging is one of the major advantages of animal imaging that cannot be done in the clinic.

My introduction to animal imaging was discouraging. I was told that the rat had to be anesthetized and that the radiofrequency signal would be received by a loop of copper wire placed at the top of the head limiting the image to the underlying cortex. With talented electrical and mechanical engineers, a company was started, aimed at designing and developing the first radiofrequency electronics and restraining systems for awake imaging in small animals.

Why animal imaging when human imaging should suffice? Indeed, structural brain imaging started in the 1980s and functional brain imaging followed in the 1990s (Ogawa et al., 1992) predating any animal studies. The answer is simple “greater experimental latitude”, i.e., we can do things in animals that we cannot do in humans. Some of the best examples can be found in the study of Schedule I drugs, i.e., drugs with no currently accepted medical use and a high potential for abuse, like methylenedioxymethamphetamine (ecstasy), heroine, and until recently, marijuana. The applications of awake imaging are the primary emphasis of this review. The section “Background” will briefly cover methodological issues around awake imaging using MRI.

## Background

### Avoiding Anesthesia

Animal imaging without anesthesia was met with some resistance, an attitude that persists today based on the many publications proposing “better” anesthetics for functional MRI studies. The contrarians argue that the reduction in stress and motion artifact using anesthesia and sedatives is better than the confound of stress when interpreting brain function. The hesitancy to adopt awake imaging is also founded in the need for specialized technology, additional costs, and time, and for some institutions, ethical issues around the care and treatment of animals. While there are multiple reasons to avoid anesthesia when studying brain function (see reviews Gao et al., 2017; Steiner et al., 2021), many obvious to the layperson, the most compelling is translation to the human condition. We do preclinical studies on brain function in animals to inform the public and guide clinical researchers in their effort to understand the human condition. This communication from bench top to clinic is made seamless if the same imaging modalities, at similar field strengths and under the same physiological conditions is performed by both. Human brain function is never studied under general anesthesia unless you are studying the effects of anesthesia. How antipsychotics, anxiolytics, analgesics, psychostimulants, drugs of abuse, etc., affect brain function cannot be studied under anesthesia. CNS active drugs affect cognition, emotion, and perception of the external and internal milieu all of which are affected by anesthesia.

### Acclimation

There are many published variations on acclimation protocols for preparing different animals for awake imaging, e.g., rats (King et al., 2005; Upadhyay et al., 2011; Russo et al., 2021), mice (Ferris et al., 2014; Yoshida et al., 2016; Madularu et al., 2017b; Han et al., 2019), marmoset monkeys (Ferris and Snowdon, 2005; Silva et al., 2011; Ziegler et al., 2020), rabbits (Wyrwicz et al., 2000), dogs (Berns et al., 2012; Cuaya et al., 2016), and rhesus macaques (Goense et al., 2010). Some describe surgical methods and implants for immobilizing the head while many use non-invasive passive restraining systems. In general, animals are lightly anesthetized with a volatile anesthetic, secured in a restraining system, and allowed to recover in a virtual environment meant to mimic the scanner. This virtual simulation with head restraint is usually accompanied by background noise mimicking the actual noise associated with the pulse sequence (Almeida et al., 2021). In our first studies (King et al., 2005) rats were acclimated for 90 min every other day for eight days. Acclimation was assessed by changes in heart rate, respiratory rate, and blood levels of the stress hormone, corticosterone. Physiologic and endocrine measures of autonomic arousal decrease with acclimation. Interestingly, when rats are imaged awake without acclimation and again after several days of acclimation we found no differences in cerebral blood flow (CBF) across multiple brain regions. Essentially baseline CBF is stable in awake rats regardless of stress, a critical point when measuring changes in functional signal dependent upon blood flow. In our more recent studies, we acclimate for five consecutive days, increasing the duration of acclimation with each subsequent day (Sadaka et al., 2021).

In some cases anesthesia can be completely avoided during the acclimation procedure. Rats surgically implanted with a head post that fits into a cradle to prevent any rotational motion can be trained over 8–10 days to enter a “snuggle sack,” slide their head post into the cradle, and lay motionless (Chang P. C. et al., 2016). While time consuming, this procedure eliminates the early use of light anesthesia required for most awaking imaging set-ups and provides excellent functional imaging with a minimum of motion artifact. Silva et al. (2011) described a step-by-step procedure for acclimating marmoset monkeys for each separate phase of the set-up, e.g., magnet environment, restraint, noise, etc., without the use of anesthesia. Ziegler et al. (2020), developed a method for acclimating preadolescent common marmoset monkeys for awake imaging without ever being exposed to anesthetics or sedatives, a condition that better reflects the human imaging experience. The unique design of the head cradle eliminated the need for ear bars or bite bar, helping to minimizing discomfort. The first study on awake dogs trained two animals to remain motionless for enough time to acquire images without the use of anesthetics, sedatives or physical restraint (Berns et al., 2012). Indeed, the absence of any exposure to anesthetics and sedatives with minimum restraint has been the case for all laboratories imaging awake dogs (Andics et al., 2014; Cuaya et al., 2016; Huber and Lamm, 2017).

The purpose of acclimation is to reduce stress and minimize motion artifact. Interestingly this is not true for all animals. We discovered that prairie voles can be imaged without acclimation on their first time in the scanner (Yee et al., 2016). Efforts to acclimate only exacerbated the stress as measured by motion artifact, vocalizations, and recording heart rate/respiratory sinus arrhythmia.

### Acoustic Noise

As noted above, MRI is noisy. The rapid changes in electromagnetic forces driving the gradients create the acoustic noise that is pulse sensitive and exacerbated at high field strengths (Mansfield et al., 1995). The problem has been effectively addressed in human imaging with hardware solutions, wave phase reduction, and altered pulse sequences, all of which can reduce the noise by as much as 20 dB (McJury et al., 1997; Hennel, 2001; Katsunuma et al., 2002). Most recently, Paasonen et al. (2020), addressed the problem in awake rat imaging by developing Multi-Band SWeep Imaging with Fourier Transformation (MB-SWIFT) at 9.4 T for EPI sequences. They report a reduction in acoustic noise by 20 to 30-dB as compared to standard EPI sequences accompanied by a decrease in motion artifact providing resting state connectivity data comparable to those reported standard EPI studies (Paasonen et al., 2020).

### Radiofrequency Coils and Restrainers

**FIGURE 1:**
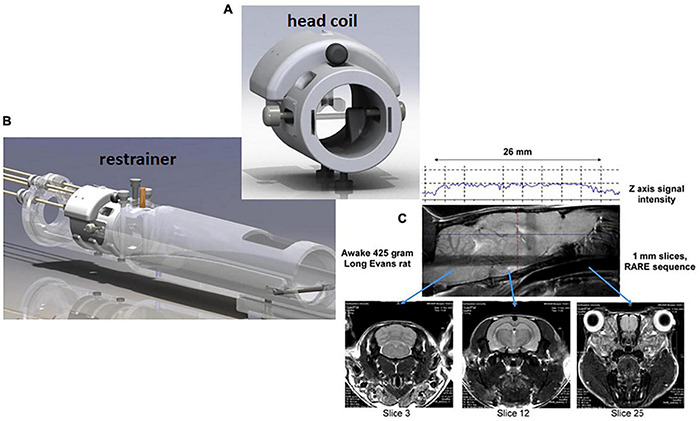
Rat imaging system. Shown are a radiofrequency coil **(A)** and restraining system **(B)** for awake rat imaging. A quadrature transmit/receive volume coil is built into the head restrainer to optimize space fitting, signal-to-noise, and field homogeneity. The design of coil allows for whole brain imaging from olfactory bulbs (slice 25) to cerebellum (slice 3) **(C)**.

Our first studies used a volume coil to transmit and a saddle shaped surface coil to receive radiofrequency signals. The head restrainer employed ear bars and a tooth bar, much like a stereotaxis, to immobilize the head. The body was restricted to a fitted tube. Since then, the RF electronics evolved into a single quadrature transmit/receive volume coil that was customized to the size of the rat’s head to optimize brain coverage and space filling for better signal-to-noise (SNR) and homogeneity (Figure 1). The ear bars were eliminated and replaced with a passive head restraining system to minimize the discomfort and stress (see video of rat setup for awake imaging https://www.youtube.com/watch?v=JQX1wgOV3K4). The system is ergonomic enabling setup in minutes, and completely non-invasive with no surgical implants used to immobilize the head. There have been several publications on the construction of RF coils and restraining systems for awake MRI imaging in rats (Martin et al., 2013; Chang J. B. et al., 2016; Madularu et al., 2017a; Stenroos et al., 2018; Derksen et al., 2021), mice (Ferris et al., 2014; Madularu et al., 2017b; Tsurugizawa et al., 2021), and the common marmoset monkey (Papoti et al., 2013, 2017; Schaeffer et al., 2019; Ziegler et al., 2020).

### Event-Related Blood Oxygen Level Dependent Imaging

**FIGURE 2:**
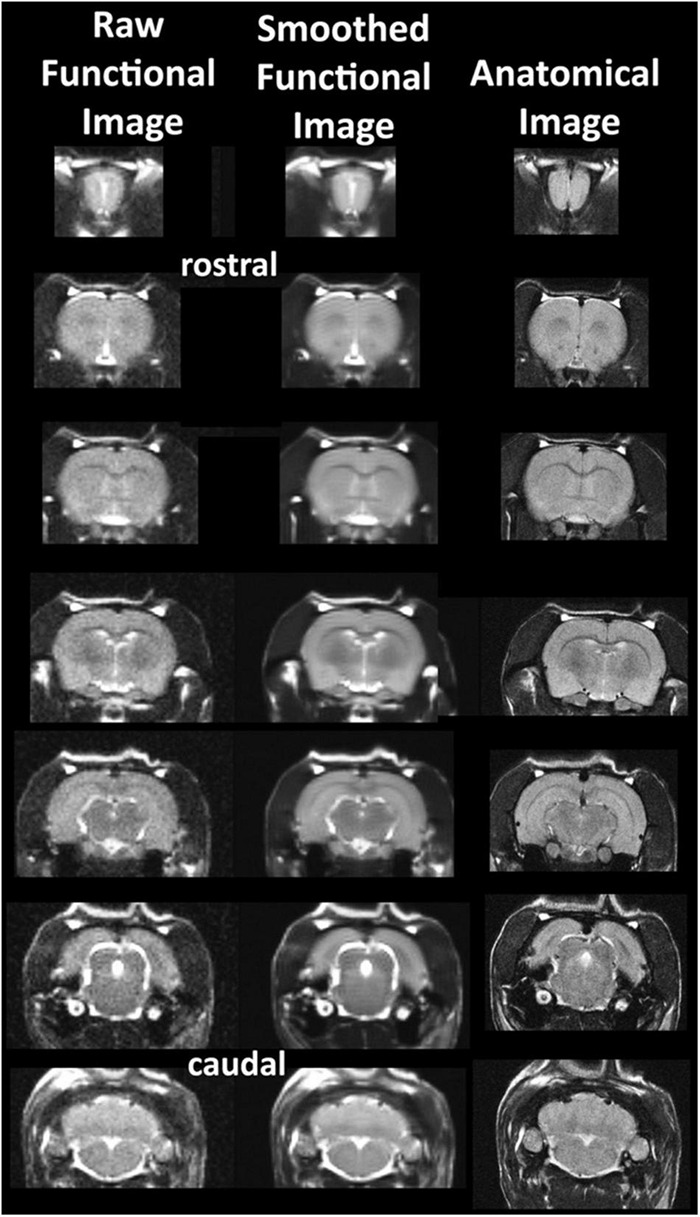
Anatomical fidelity. Shown are examples of brain images collected during an awake imaging session in rats using a multi-slice spin echo, RARE (rapid acquisition with relaxation enhancement) pulse sequence. The anatomical images to the right are taken at the beginning of the scanning session. The column on the left shows the same images but collected for functional analysis using HASTE, a RARE pulse sequence modified for faster acquisition time. Whole brain acquisition time is approximately six sec with a spatial resolution of 312 μm^2^. The images in the middle column have been smoothed during pre-processing. Note the anatomical fidelity between the functional images and their original anatomical image. The absence of any distortion is necessary when registering the data to an atlas. (Adapted with permission from Ferris et al. (2015)).

The common read-out for awake imaging in animals and humans is a change in blood oxygen level dependent (BOLD) signal contrast (Kim and Ogawa, 2012) as compared to a baseline obtained prior to the introduction of a drug or a general stimulus. The change in signal can occur within 3–10 s after provocation and can persist for a different duration in time dependent upon the experimental paradigm and the presence or absence of anesthesia (Martin et al., 2006). In some of the earlier studies changes in CBF and contrast enhanced cerebral blood volume (CBV) were measured as markers for functional brain activity. To optimize anatomical fidelity a spin echo pulse sequence can be used. It should also be emphasized that high neuroanatomical fidelity and spatial resolution are critical in identifying distributed neural circuits in any animal imaging study. Many brain areas in a segmented rat atlas have in-plane boundaries of less than 400 μm^2^ and may extend for over 1,000 μm in the rostral/caudal plane. With the development of a segmented, annotated 3D MRI atlas for rats (Ekam Solutions, Boston, MA, United States), it is now possible to localize functional imaging data to 173 precise 3D “volumes of interest” in clearly delineate brain areas. Therefore, it is critical that the functional images are a very accurate reconstruction of the original brain neuroanatomy as shown in Figure 2.

### Resting State Functional Connectivity

This modality was originally described by Biswal and colleagues as connections between brain areas based on similar low frequency fluctuations in BOLD signal (Biswal et al., 1995). Realistically, resting state in awake animal imaging means no external stimulus. There is undoubtedly some level of stress associated with resting state functional connectivity (rsFC) in animals, particularly rodents. However, following different acclimation protocols, rsFC neural circuits analogous to those reported in human imaging have been identified in animals (for recent review see Pais-Roldan et al., 2021). The first method for rsFC in awake rats was published by Zhang et al. (2010) using a seed-based correlation analysis. Subsequently, studies from different labs used independent component analysis to identify functional clusters or neural circuits, e.g., default mode, salience mode, analogous to those found in humans (Becerra et al., 2011b; Liang et al., 2011; Upadhyay et al., 2011; Belcher et al., 2013; Liu et al., 2019). Tu et al. (2021) combined Designer Receptors Exclusively Activated by Designer Drugs (DREADDs) chemistry and rsFC to suppress the anterior cingulate cortex, a key circuit hub, and showed alterations in whole brain neural circuit organization and integration in the rat. Combining optogenetics with rsFC, Desai et al. (2011), showed they could activate the primary somatosensory cortex and recruit a distributed neural circuit of brain regions that included the motor cortex, striatum, and contralateral somatosensory cortex in mice. rsFC in mice has also been used to identify individual variations in the organization of the cortex that could predict differences in motor behavior (Bergmann et al., 2020). However, there is a dilemma when imaging rsFC in awake animals as noted above. How to choose between the confound of mild stress and arousal associated with the fully awake condition or the use of light anesthesia or sedation which would reflect a true “resting” condition, albeit artificial. The BOLD signal obtained with a combination of isoflurane and medetomidine, an α2 adrenergic receptor agonist, is reported to accurately reflect changes in brain activity and associated neural vascular coupling for use in rsFC (Grandjean et al., 2014; Brynildsen et al., 2017). However, there have been other studies comparing different anesthetics that do not support the use of isoflurane/medetomidine as the best choice for rsFC (Paasonen et al., 2017, 2018), a disparity possibly due to the dosing regimen of medetomidine (Kint et al., 2020).

### Small Animals

Over time there have been several publications across numerous mammalian species on methods and applications for awake imaging. These include mice (Ferris et al., 2014), rats (Febo, 2011), voles (Yee et al., 2016), rabbits (Weiss et al., 2018), cats (Ma et al., 2013), dogs (Berns et al., 2012), pigeons (Behroozi et al., 2020) common marmosets monkeys (Ferris et al., 2001; Papoti et al., 2013; Silva, 2017), and rhesus macaques (Goense et al., 2010). Each of these species, most obviously rhesus monkey, have general or unique attributes that provide a better understanding of the human condition. For example the special relationship between dogs and humans and the communication between them [see seminal studies by Andics et al. (2014, 2016) and Huber and Lamm (2017), Karl et al. (2020, 2021)]. However, much of the work today is focused on rodents. From the early CSI-II 2.0T produced by GE to the ultra-high field 11.7T system developed by Bruker, the bore size was kept small to reduce the cost of the technology while pushing the limits of spatial resolution. Hence these small-bore systems (≤30 cm) favor the use of small animals. To that point, this review is primarily focused on rodents and their many applications in awake imaging.

## Applications

### Pharmacological MRI

One of the major applications of awake imaging in rodents has been driven by pharmaceutical companies to aid in characterizing or “fingerprinting” drug candidates in early drug discovery. Does a drug candidate get into the brain and interact with its target in a dose-dependent manner? The general protocol is simple and usually involves three different doses of drug plus a vehicle control. These are usually small molecules with specific CNS targets. Scientists from Abbott Laboratories (Abbott Park, IL, United States) pioneered the work on Pharmacological MRI (phMRI) in awake rats. Using a potent agonist for α4β2 nicotinic acetylcholine receptor they mapped the change in brain activity that fit the know localization of the receptors demonstrating target engagement and activation of neural circuitry that fit the behavioral data (Skoubis et al., 2006). In another study they used phMRI to show dose-dependent activation of neural circuitry involved in emesis as a model for predicting emetic liability of early drug candidates (Chin et al., 2006). Addressing the topic of pain regulation through cannabinoid signaling they used a non-selective CB1/CB2 and selective CB2 receptor agonists to affect BOLD signal and concluded CB1 and not CB2 was the primary target of cannabinoid signaling (Chin et al., 2008). They also mapped the BOLD activation pattern of subanesthetic doses of ketamine showing both positive and negative BOLD changes over the brain (Chin et al., 2011). These studies included drugs that would block the effects of ketamine as a potential imaging method for screening new anti-psychotics (Baker et al., 2012). Tang et al. (2018) used phMRI to map brain activation in awake rats in response to ketamine and traxoprodil, a putative antidepressant and selective GluN2B NMDA receptor antagonist. Traxoprodil produced a dose-dependent change in specific brain regions many that overlap with the activation pattern of ketamine (Tang et al., 2018).

In a just published study in mice treated with the phytocannabinoid cannabidiol (CBD) we reported a dose-dependent polarization of BOLD signal change along the rostral-caudal axis of the brain with the forebrain showing positive signal while the brainstem and cerebellum presented with negative signal (Sadaka et al., 2021). The brainstem ascending reticular activating system (ARAS) was decoupled to much of the brain but was hyperconnected to the olfactory system and prefrontal cortex. In one of the more compelling phMRI studies, Becerra et al. (2013) ran a parallel experiment between rats and humans comparing species equivalent doses of buprenorphine, a partial mu-opioid receptor agonist on circuit activation and showed comparable patterns of BOLD signal change. This study demonstrated the significance of phMRI in translating drug function between rats and humans in early drug discovery. In a subsequent study, published only a year later, this translation of buprenorphine activity across species using phMRI was also demonstrated using cynomolgus monkeys (Seah et al., 2014).

Not all small molecules with CNS targets present with a dose-dependent change in brain activity. To this point, is evidence from our lab on a study using the neuropeptide oxytocin, a neurochemical signal involved in social and emotional behavior (Carter et al., 2020). Systemically administered oxytocin failed to induce a dose-dependent change in BOLD signal in a study comparing central vs peripheral routes of oxytocin administration (Ferris et al., 2015). While a single dose of intracerebroventricular (ICV) oxytocin activated many of the brain areas with a high density of oxytocin receptors, peripheral oxytocin failed to do so. However, peripheral oxytocin had its own unique “fingerprint” affecting the olfactory bulb, cerebellum, and several brainstem areas relevant to the ARAS. The patterns of brain activity suggest that peripheral oxytocin may interact at the level of the olfactory bulb and, through sensory afferents from the autonomic nervous system, influence brain activity This raises an interesting consideration when performing any functional brain imaging study in response to a systemically administered drug. While the drug may cross the blood brain barrier and show target engagement, the final profile of BOLD activation may have contributions from the peripheral nervous system.

### Drugs of Abuse

#### Cocaine

In the early 2000s, Febo and colleagues published a series of foundational studies on the effects of cocaine on brain function in awake rats. To avoid perturbations in cardiovascular and respiratory function they used ICV injections during the scanning session to elicit activity in dopaminergic pathways (Febo et al., 2004). To follow the neuroadaptive changes that occur with drug addiction, they treated rats for several consecutive days with cocaine followed by a washout period and cocaine challenge during awake imaging (Febo et al., 2005c). Rats with a history of cocaine exposure showed a blunted BOLD response in dopaminergic pathways as compared to drug naïve rats. Following up on the literature reporting estrogen alters behavioral sensitivity to cocaine (Becker, 1999), Febo et al. (2005a) worked with ovariectomized rats with and without estrogen treatment and chronically exposed to cocaine. Following a washout period, females with estrogen showed enhanced BOLD signal changes in dopaminergic pathways as compared to drug naïve controls exposed to cocaine in contrast to the male response reported in their previous study. The Febo lab continued to publish numerous studies on cocaine in awake rats on topics that included sexual receptivity (Febo et al., 2011), odor processing (Johnson et al., 2013; Caffrey and Febo, 2014), epigenetics (Febo et al., 2009a), and maternal care in rats addicted to cocaine prior to pregnancy (Febo and Ferris, 2007; Nephew et al., 2012).

**FIGURE 3:**
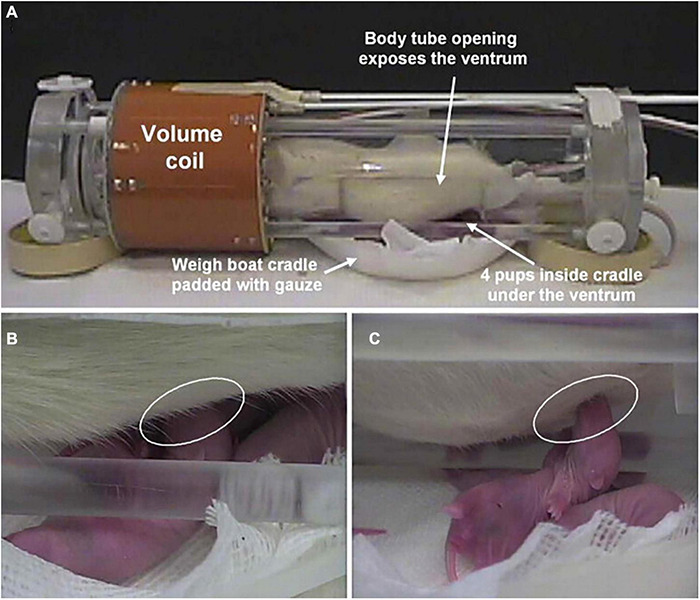
Setting for pup suckling in the magnet. Shown is the experimental setup for imaging pup suckling during the scanning session of awake dams. **(A)** The opening in the body tube allowing pups to gain access to the dam for nursing. Pups are in a cradle beneath the dam. **(B)** Pup suckling is established within seconds during a functional scan. **(C)** Side view showing a suckling pup. (Adapted with permission from Febo et al. (2005b)).

Using a modified rat holder that enabled dams to breast feed during the imaging session (Figure 3), we asked the question “Is pup suckling more rewarding than cocaine?” (Ferris et al., 2005). Before the imaging session, 4-to-8-day old pups were physically isolated from their mother in the home cage for 2–3 h by confining them to an inverted perforated Plexiglas box. These pups were then positioned in the magnet in a cradle beneath the mother separated by a thin plastic sheet. During the imaging session the sheet could be removed exposing the hind end teats, at which time the pups would immediately start to feed. Pups sucking induced a robust positive BOLD activation of dopaminergic circuitry with little negative BOLD. In the same study, virgin females given cocaine showed the same pattern of positive BOLD activation with little negative BOLD evidence that both breast feeding and cocaine elicit similar brain mechanisms regarding motivation and reward. Interestingly, when mothers were exposed to cocaine and not their pups the pattern of brain activity was reversed - the dopaminergic reward circuitry showed primarily negative BOLD. Hence, the reproductive condition of the female appears to be a critical determinant in predicting the effect of cocaine on brain activation.

#### Opioids

Kaufman et al. (2013) published the first study on opioids using fentanyl a mu receptor agonists and U69,593 a kappa agonist in awake cynomolgus monkeys and showed selective activity for each drug on reward circuitry. The activity of oxycodone was characterized in a genetic mouse model devoid of the mu-opioid receptor vs wild type controls (Moore et al., 2016). The positive BOLD signal change essentially mirrored the localization of mu-opioid receptors in wild-type but was absent in the knock-out mice when mapped onto a segmented 3D MRI atlas with 122 brain areas. The pattern of activity in wild-type mice was also closely associated with key brain areas involved in pain regulation. The effects of acute and chronic exposure to oxycodone in rats were studied to follow the neuroadaptive changes that occur with opioid addiction (Iriah et al., 2019). The initial design had drug naïve rats imaged during for their first scanning session in response to systemically injected oxycodone. Rats were then treated on four consecutive days with oxycodone in a conditioned place preference chamber to demonstrate drug seeking behavior. On the following day rats were again scanned in response to vehicle control or oxycodone. The study was terminated because of severe motion artifact during the baseline acquisitions for both treatments. Interestingly, the hyperexcitability was ascribed to withdrawal symptoms as treatment with oxycodone eliminated the motion artifact that persisted with vehicle. To follow the neuroadaptive changes that occur with chronic oxycodone exposure it was necessary to use manganese enhanced imaging with general anesthesia to localize the accumulation of contrast agent in dopaminergic pathways, hippocampus, and amygdala.

#### Amphetamine

Madularu et al. (2015b) studied brain activity in ovariectomized female rats sensitized to amphetamine with different levels of estrogen replacement and in the face of a haloperidol treatment. Amphetamine on a background of high estrogen enhances brain activation in dopaminergic pathways (Madularu et al., 2015b). Ovariectomized rats exposed to daily treatments of the antipsychotic haloperidol together with estrogen replacement also show enhanced responsivity to amphetamine challenge in neural circuits associated with schizophrenia, e.g., hippocampus, habenula, amygdala, and alterations in whole brain and hippocampal volumes (Madularu et al., 2015a,^2016^). Together these studies suggest awake imaging in rats can be used to assess the interaction between ovarian steroids and the sensitivity of neuroleptics to impact dopaminergic signaling in brain areas associated with schizophrenia. Awake rats challenged with amphetamine show an increase in cerebral blood volume correlated with changes in striatal dopamine concentrations (Kashiwagi et al., 2015).

#### Tetrahydrocannabinol

**FIGURE 4:**
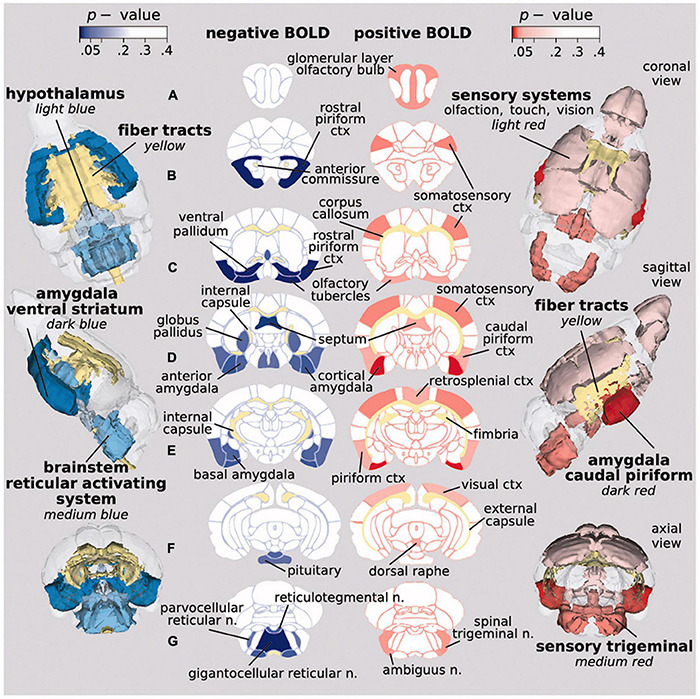
Activation maps on inhaled vaporized cannabis. Probability maps showing significant changes in BOLD signal in brain areas that differed in mice exposed to inhaled cannabis or placebo. Positive BOLD (red) reflects an increase in signal, while negative BOLD (blue) indicates a decrease. (Adapted with permission from Farra et al. (2020)).

Tetrahydrocannabinol (THC), the psychoactive ingredient in cannabis, was first tested in awake rats by systemic injection (Madularu et al., 2017c). Low dose of THC, but not vehicle or high dose THC increased positive and negative BOLD signal in areas high in CB1 receptors and in neural circuits associated with pain and cognition. Fara and coworkers ran the first and only imaging study to date on awake rodents looking at changes in brain activity in drug naïve mice exposed to vaporized cannabis (12.3% THC) during the scanning session (Farra et al., 2020). This study using inhaled cannabis was designed to achieve blood levels of THC reported in human recreational use via a route of administration that better reflects the human experience. There were robust changes in both positive and negative BOLD signal (Figure 4). The positive BOLD activation map suggests enhanced sensory processes while the negative BOLD map suggests reduced behavioral arousal.

#### MDMA

The studies on methylenedioxymethamphetamine (MDMA, ecstasy) in awake animals are few. Because MDMA is a Schedule I drug there are no studies on drug naïve humans. During the scanning session an oral dose of MDMA was given to marmosets and images acquired over 90 min (Brevard et al., 2006b; Meyer et al., 2006). The change in brain activity over time was correlate with the change in blood levels of MDMA.

#### Nicotine

Li et al. (2008) studied the addictive properties of nicotine in awake rats by comparing the change in BOLD signal intensity to acute and chronic drug exposure. Repeated exposure to nicotine prolongs BOLD activation in the hippocampus, reward circuitry and prefrontal cortex. In a subsequent study, varenicline, a partial agonist at the α4β2 nicotinic acetylcholine receptor used to treat nicotine addiction, was tested in awake rats, and was shown to produce a pattern of brain activity not unlike nicotine alone. However, repeated exposure to varenicline failed to cause the sensitization effect of nicotine (King et al., 2011) evidence in support of varenicline’s therapeutic efficacy. Adolescent male rats chronically exposed to nicotine with or without menthol show differences in functional connectivity (Thompson et al., 2018). Menthol combined with nicotine show enhanced functional connectivity in reward circuitry and other regions not seen with nicotine alone.

#### Ethanol

There are only two studies done on awake rats given ethanol during the scanning session. The first measured the change in CBV using contrast enhanced functional MRI and reported a robust increase in activity in cortex and striatum (Luo et al., 2007). The second, trained rats to respond to the conditioned stimulus of a light pulse during imaging while simultaneously giving i.p. ethanol. After several days of this conditioning procedure, the caudate-putamen, anterior insular cortex, hippocampus, ventral pallidum, and nucleus accumbens were activated by light alone (Tsurugizawa et al., 2012).

### Sensory Evoked Stimuli

There have been many imaging studies on awake animals following changes in brain activity to natural stimuli like olfaction, visual cues, sound, touch, and taste. Mice given light pulses of different durations and frequencies showing activation in visual pathways (Dinh et al., 2021). The most common stimulus in rodents is the presentation of an odor. Kulkarni et al. mapped changes in brain activity to four different odors presented to rats during the scanning session (Kulkarni et al., 2012). As expected, all odors activated olfactory pathways; however, one odor, benzaldehyde, the smell of almond, increased activity in brain areas associated with emotion, memory, and reward. Indeed, mammals have genes that code for the benzaldehyde receptor in olfactory epithelium (Bozza et al., 2002). That evolution would favor the smell of almond is not surprising given its caloric density and durable structure. Hence the smell of almond elicits a positive emotional response in rats. What makes this study so interesting is that rats challenged with benzaldehyde were “odor naïve” never having been exposed to almond. The response was innate! The brain is wired to recognize benzaldehyde as a rewarding stimulus. As a validation for awake imaging in prairie voles, animals were presented with different odors and the change in brain activity mapped to a 3D MRI vole atlas (Yee et al., 2016). Zhifeng and coworkers developed an awaking imaging system in mice that allows behavioral responses, e.g., licking, to odor cues (Han et al., 2019). Using an implanted head holder, mice can be trained to lick for water during in a go/no-go conditioning paradigm driven by odor cues. This same awake mouse imaging system can also be used to follow brain activity in response to sensorimotor and auditory stimuli during the scanning session (Chen et al., 2020). Male marmoset monkeys presented with the smell of sexual receptive females show positive BOLD activation in many areas of the brain (Ferris et al., 2001) that is reversed, i.e., negative BOLD, when the stimulus comes from an ovariectomized female (Ferris et al., 2004).

With respect to marmoset monkeys, there are awake imaging studies mapping brain activity in response to touch, sound, and visual stimuli. The application of light touch by a puff of air delivered to the face, arm or leg produces cortical and subcortical maps of topographical body representation (Clery et al., 2020a). Presentation of mixed and pure tones revealed the auditory cortex has tonotopic organization for processing complex and simple stimuli (Toarmino et al., 2017). There are several studies looking at the response of the visual cortex and associated neural circuitry to various types of visual stimulation paradigms. Marmosets can be taught to carry out visual tasks during the scanning session in response to the presentations of different images, e.g., conspecific faces, conspecific body parts and general objects, resulting in the activation of visual cortex and subcortical areas (Hung et al., 2015b). The regional pattern of activity is selective for different categories of visual stimuli. For example, the occipitotemporal pathway shows the strongest preference for faces over other stimuli (Hung et al., 2015a). Objects in motion, coming toward or receding, evoke activity across a large cortical network involving frontal, parietal, temporal, and occipital areas. The activation is greatest as objects come closer suggesting a network for processing stimuli entering a peri personal space (Clery et al., 2020b). Marmosets can be imaged while watching movies to record patterns of brain activity that reflect a more naturalistic, complex stimulus. Patterns of brain activity in movie driven fMRI between marmoset monkeys and humans show similar functional correlates and circuit organization (Hori et al., 2021).

The original “vivarium” studies included the resident/intruder model (Ferris et al., 2008), pup suckling model (Ferris et al., 2005; Febo et al., 2008) (see Figure 3) and another imaging lactating dams responding to the visual presentation of their own pups with and without a male intruder (Nephew et al., 2009). This staged environmental situation using other animals to elicit changes in brain activity was recently used by Gilbert and coworkers to model social interactions between marmosets by simultaneously measuring brain activity in awake marmosets during the scanning session (Gilbert et al., 2021). Each restraining system was equipped with its own RF electronics. Each monkey was positioned facing each other providing the means for visual and olfactory communication. The activation maps generated by this social interaction were associated with visuomotor tasks.

### Brain Disorders

#### Epilepsy

In a series of seminal studies, Ferris and Tenney (2013) used awake imaging in rats and marmoset monkeys to study the neural circuitry of absence seizures. Absence seizure or petit mal do not involve uncontrolled skeletal muscle contraction; instead, the seizures is characterized by a 3 Hz spike wave discharge on EEG accompanied by a trance-like behavior that may persist for over a min (Snead, 1995). Without the confound of motion artifact this type of epilepsy is fertile ground for awake imaging. Giving rats γ -hydroxybutyric acid (GBH), a metabolite of the inhibitory neurotransmitter GABA, Tenney et al. (2003), could triggered an absence seizure during the scanning session. Unlike humans the slow wave discharge is 6–7 Hz s; nonetheless, the pattern of brain activation was centered on corticothalamic neural circuitry fundamental to human absence seizures. In a subsequent study using the WAG/Rij rat, a genetic model of spontaneous absence seizure, Tenney et al., used the onset of the 6-7 Hz EEG signal to initiate image acquisition (Tenney et al., 2004a). This EEG-triggered BOLD imaging shows a dominant corticothalamic activation pattern similar to the GBH-induced seizure. In a final study they used the precursor molecule to GBH, γ-butyrolactone (GBL), to induce absence seizures in marmoset monkeys (Tenney et al., 2004b). In this model they were able to reproduce the 3Hz spike wave discharge, the diagnostic criteria for a human absence seizure, making this model more relevant to the human condition. Again the absence seizure is dominated by corticothalamic activation that could be blocked by treatment with the antiepileptic drug, ethosuximide. While it is not possible to image a generalized tonic-clonic seizure due to motion artifact, it is possible to image the organization of brain activity that contributes to the genesis of this type of seizure. To this end, Brevard et al. (2006a) used pentylenetetrazol (PTZ) to trigger a tonic-clonic seizure in rats during the scanning session. Within seconds of giving PTZ ICV there is an increase in BOLD signal in the thalamus, particularly anterior thalamic nucleus, retrosplenial ctx and hippocampus. These areas peak in BOLD activation just seconds before seizure onset. Pretreatment with ethosuximide blocks all activity in the anterior thalamic n. and retrosplenial cortex and prevents seizure onset.

#### Fragile X Syndrome

The olfactory stimulus of almond that triggers the innate circuitry involved in motivation toward a rewarding stimulus was tested in a genetic rat model of Fragile X (Kenkel et al., 2016). Fragile X syndrome is the leading genetic cause of autism, a condition noted for withdrawal, gaze aversion and disrupted social communication. The study was done with “odor naïve” wild-type and fragile X mental retardation 1 gene (fmr1) KO male rats. When exposed to the smell of almond, wild-type rats show the expected activation of olfactory and reward circuitry. KO rats on the other hand, fail to show positive BOLD activation, but instead show negative BOLD in brain areas associated with emotion and reward. These findings suggest that the disrupted social communication and avoidance behavior in autism may be due, in part, to the inability to process rewarding stimuli.

#### Huntington’s Disease

The presentation of almond odor has also been used to map brain activity in a mouse model of Huntington’s disease, the zQ175 knock-in mice containing a human mutant allele with the expanded CAG repeats within the native mouse huntingtin gene (Ferris et al., 2014). Changes in BOLD activity were compared between wild-type, HETzQ175, and HOMzQ175 genotypes. HOMzQ175 mice show a reduced response to almond odor as compared to the other genotypes suggesting a deficit in olfaction which is common in neurodegenerative diseases (Patino et al., 2021). The expected changes in cortex and basal ganglia that characterize Huntington’s were not observed; instead, there was gene-dose effect on brain areas high in huntingtin associated protein 1.

#### Alzheimer’s Disease

Sakurai et al. (2020) probed the APPswe / PSEN1dE9 double transgenic mouse model of Alzheimer’s for biomarkers of altered reward-based behavior. To do so, they developed an imaging system to follow changes on BOLD activity during licking behavior in a 14T scanner. Thirsty mice are given light cues to elicit drinking. The conditioned drinking response is associated with hyperactivity in the raphe nucleus and hippocampus, a response that may reflect the enhanced impulsivity common to Alzheimer’s.

#### Parkinson’s Disease

While this review is primarily focused on small rodents and marmoset monkeys, attention should be drawn to the work of Zhang et al. (2000) on their studies using awake rhesus monkeys to understand drugs that impact basal ganglia activity in normal controls, during aging (Zhang et al., 2001) and in a MPTP model of Parkinson’s disease (Zhang et al., 2006). Studies combining normal aging with aging plus MPTP and drug treatments involving the dopamine receptor agonist apomorphine and different dopamine receptor antagonists were able to parse out the altered motor dysfunction seen in Parkinson’s and aging as changes in striatum and globus pallidus, respectively (Andersen et al., 2015). The results show it is feasible to use drug provocation protocols to differentiate Parkinson’s from age-associated parkinsonism.

### Pain

Studies on pain necessitate the use awaking imaging. The data collected from acute and chronic pain models provide insights into the integrated neural circuits processing nociceptive stimuli and the neuroplasticity of these circuits to chronic pain and their responsiveness to therapeutics. Becerra et al. (2011a) applied a thermal stimulus of 48°C to the hindpaw of awake rats and reported activation of cortical, subcortical and brainstem areas consistent with the know literature on pain neural pathways. These researchers also studied a unique rat model for migraine pain that entails the application of a cocktail of inflammatory agents to the dura of awake rats to activate nociceptive fibers in the meninges (Becerra et al., 2017). This inflammatory soup (IS) model was used to look at the altered sensitivity to mechanical and thermal stimulation and resting state FC during the scanning session. With this model of intracranial pain they found hypersensitivity to mechanical stimulation of the face as evidenced by an increase in BOLD signal in hypothalamus, hippocampus, and somatosensory cortex with altered rsFC. In a subsequent study sumatriptan, 5HT-1B/D-receptor agonist and naproxen sodium, a COX-1 and COX-2 inhibitor, drugs used to treat migraines were tested in the IS model and shown to alter whole brain neural circuits that included the default mode, salience, sensorimotor, and cerebellar networks (Bishop et al., 2019). The IS model has also been used by others to show altered rsFC in the periaqueductal gray and anterior cingulate to brain regions involved in the sensory and emotional aspects of migraine headaches (Jia et al., 2017, 2019). Yee et al. (2015) looked at the response to intradermal capsaicin injection into the hindpaw of rats. The specificity of the pain response to capsaicin was tested in a transgenic rat model with a deletion of the TRPV1 gene, the target of capsaicin. The putative neural circuitry of pain is activated in wild-type but not transgenic rats. In addition, wild type but not KO rats show activation of Papez (Kulkarni et al., 2012) and habenular neural circuitry both of which play important roles in emotional experience, learning and memory of aversive information. Awake imaging has been used to test and demonstrate the efficacy of a DALDA analogue, a potent mu-opioid peptide delivered as an encapsulated nanoparticle on capsaicin-induced pain (Shah et al., 2014). Chang et al. (2017) showed the development of neuropathy was associated with changes in BOLD activity in prefrontal ctx and accumbens. With respect to awake imaging and pain it should be noted that the process of acclimating animals to the stress of restraint may affect the pain response, a point of concern that should be considered when interpreting the data (Low et al., 2016).

### Social Behavior and Fear

#### Oxytocin and Affiliation

Oxytocin and vasopressin are two phylogenetically old neuropeptides found throughout the animal kingdom and co-opted by different species for numerous functions primarily related to social behavior (Ferris, 2008). Oxytocin has been associated with affiliation and pair bonding while vasopressin with approach/avoidance and aggression. During imaging, Febo and colleagues exposed dams to pup suckling (see Figure 3 section on Drugs of Abuse) before and after ICV administration of an oxytocin receptor antagonist while another group of dams was given ICV oxytocin alone (Febo et al., 2005b). Pup sucking and exogenous oxytocin cause similar patterns of positive BOLD activation in brain areas associated with olfaction, emotions, motivation, and reward while blockade of oxytocin receptors reduces this activation. These data add to a body of literature that oxytocin may strengthen mother–infant bond formation. In a subsequent study lactating dams were tested for fear elicited by exposure to predator scent trimethylthiazoline (TMT) the smell of fox presented during the imaging session (Febo et al., 2009b). Pretreatment with oxytocin alters the innate fear response to threat of predation, enhancing brain activity in areas associated with emotion and cognition and reducing activity in areas involved in autonomic visceromotor and skeletomotor responses.

In a study testing central vs peripheral administration of oxytocin, Dumais et al. (2017) looked for differences in brain activity in awake male and female rats. Males are more responsive to both centrally and peripherally administered oxytocin than females. Centrally administered neuropeptide activates areas associated with motivation and reward. However, peripheral oxytocin modulates neural activation differently in male and female rats. The pattern and magnitude of activation are different, but not the direction, showing a sex differences depending on the route of administration. These findings highlight the need to include both sexes in basic and clinical studies to fully understand the role of oxytocin on brain function.

#### Vasopressin and Aggression

There are numerous studies on the role of vasopressin as a chemical signal in the brain controlling agonistic behavior (for review see Febo and Ferris, 2014). Giving vasopressin or a selective arginine vasopressin, V1a receptor antagonist can enhance or inhibit aggressive responding, respectively (Ferris et al., 1999, 2006). Similarly, there are many studies showing that serotonin (5HT) receptor agonists and reuptake inhibitors like fluoxetine reduce aggressive behavior (e.g., Ferris et al., 1997, 1999).

Since overt aggression in a laboratory setting is measured by physical bites and attacks on a conspecific it does not lend itself to awake imaging. However, aggressive motivation, i.e., the autonomic arousal preceding overt aggression can be measured. Aggressive motivation can be elicited by putting a male intruder into the home cage of a resident male and his female partner. In this context, the resident male will attack the intruder. The intruder is forewarned of the impending attack by the piloerection of fur along the back of the resident. The piloerection is an autonomic display of a centrally organized response of aggressive motivation or intent to attack. Giving the resident male an orally active, selective V1a receptor antagonist or a 5HT reuptake inhibitor will block piloerection toward an intruder. This resident/intruder paradigm with piloerection can be staged in the magnet during the scanning session (Ferris et al., 2008). This is done with a “vivarium,” a housing unit containing bedding from the home cage along with the resident’s female partner. The resident is prepared for awake imaging and positioned in the magnet. The vivarium is then positioned in the magnet centimeters away from the resident’s head. Five minutes into data acquisition, an intruder is placed into the vivarium. Within less than a min the resident will piloerect. In this study the change in brain activity was mapped to a 3D MRI rat atlas with over 100 brain volumes. The increase and decrease in activity across numerous brain areas correlates with the piloerection and is interpreted to be the neurocircuitry of aggressive motivation. Both drugs block key nodes in the known neural circuitry of aggression.

#### Fear

The first study on fear in awake animals was done in the Flinders Sensitive Line (FSL), a rat model of depression. Trimethylthiazoline was used to trigger the innate fear of predation in FSL and control rats causing differences in activity in the prefrontal cortex and amygdala (Huang et al., 2011). Holmes and colleagues followed with a series of studies in rats and mice using a conditioned-fear model associating a light cue with foot shock (Brydges et al., 2013; Harris et al., 2015, 2016). This conditioned-fear response was performed during the scanning session and the change in BOLD signal mapped to fear neural circuitry. This study was repeated again but with rats having a history of early life stress. Early life stress in rats produces depression-like behavior. With this model the conditioned fear response is exacerbated as compared to controls. In a subsequent study, rats deficient in brain-derived neurotrophic factor (BDNF), a chemical signal associated with psychiatric disorders, were tested for conditioned fear during imaging (Harris et al., 2016). Interestingly these rats fail to activate fear circuitry as compared to controls showing a functional relationship between BDNF and emotional regulation. Awake imaging can also be used to image condition taste aversion. During an imaging session the sweet taste of saccharine can be associated with the nauseating effect of i.p. lithium chloride. The BOLD signal response shows activation of prelimbic ctx, insular ctx and basolateral amygdala. This neural circuitry was confirmed by connectivity studies using diffusion tensor imaging and postmortem histology and c-Fos for cellular activation (Uematsu et al., 2015). Rats can also be conditioned to associate the taste of sucrose with the single exposure of a live predator, the sable ferret, during a scanning session. This initial exposure elicits the expected activation of emotional and cognitive neural circuits. However, when reimaged two weeks later the increase in brain activity in fear circuitry in response to the taste of sucrose alone is exacerbated suggesting the memory of the initial aversive experience is worse than the experience itself (Ferris et al., 2011). A single exposure to predator odor can alters rsFC in fear circuitry. Rats exposed to the collar taken from a cat vs an unworn collar, display heightened levels of anxiety one week later and alteration in connectivity between the infralimbic ctx and amygdala (Liang et al., 2014).

Any experience associated with high stress like predatory fear engages the brain-adrenal axis and the release of glucocorticoids—stress hormones. How much of the behavioral response, conditioned or innate, is affected by adrenal steroids? It is well known that glucocorticoids can have long term effects by acting like transcription factors affecting gene activity and neural plasticity (Kim and Diamond, 2002). Can stress hormones have an immediate non-genomic effect altering the behavioral response to an aversive emotional experience? To address this question, blood levels of corticosterone were measured in rats exposed to low and high stress conditions (Ferris and Stolberg, 2010). During a scanning session, adrenalectomized rats were given i.v. injections of corticosterone that elevated blood levels to those measured in intact rats. Within the first 2 min there was a significant increase in brain activity in the neural circuitry of emotion and memory to the high stress glucocorticoid injection but not the low stress injection showing that stress hormones can have immediate non genomic effects on brain activity. A thorough review on stress and functional MRI in humans and animals is provided by Dopfel and Zhang (2018).

### Novel Applications

#### Brain Clearance

**FIGURE 5:**
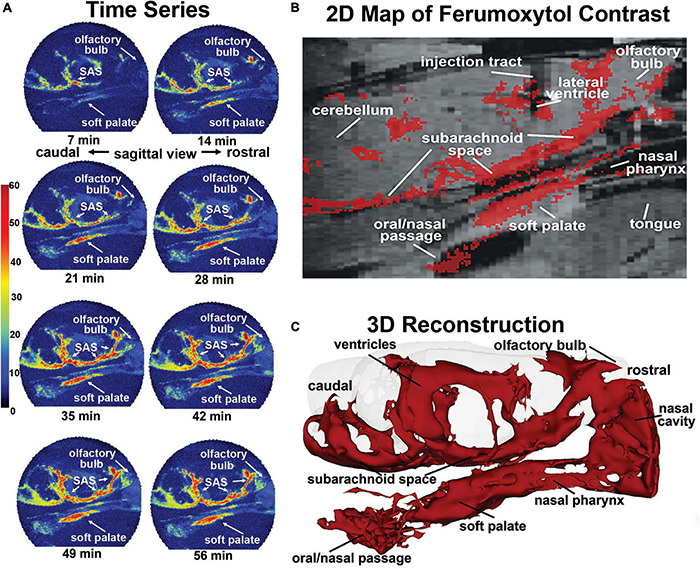
Mapping clearance from brain to soft palate. Shown is the movement and accumulation of ferumoxytol. The time series **(A)** are from a single subject, collected at 7-min intervals over 70 min long scanning session. A summary image **(B)** of the average CA signal from six rats is mapped onto the T2 weighted anatomy image of the brain. **(C)** The 3D reconstruction of CA within and outside the brain from a single subject at the end of the imaging study. (Adapted with permission from Leaston et al., 2021).

Much has been written about the glymphatic system and clearance of metabolic waste and unwanted proteins and the confound of anesthesia (Ferris, 2021). ICV injected tracer into awake mice rapidly appears in the lymphatic system and systemic circulation (Ma et al., 2017), but is retarded in the presence of anesthesia (Ma et al., 2019). When imaging is performed during the dark phase of the mouse light-dark cycle, a time when they are most active and clearance is low, ICV tracer rapidly comes to equilibrium across the brain parenchyma under awake conditions but not under anesthesia (Gakuba et al., 2018). Cai et al. (2020) published the first study on the circadian control of perivascular clearance altering the time of image acquisition between the light and dark phases of a 24 h cycle. ICV injection of MRI contrast agent in awake rats effectively moves throughout the brain parenchyma during the dark phase but not so during light when rats are at rest or sleeping. This pattern of trace accumulation indicates greater clearance during light phase than the dark phase. Mild repetitive head injury without evidence of brain damage alters perivascular clearance in the midbrain dopaminergic system weeks after insult. This was shown by awake imaging of MRI contrast agent in rats during the light phase of the circadian cycle (Cai et al., 2021). Perivascular clearance of ferumoxytol an iron oxide MRI contrast agent infused ICV in awake rats during the circadian light phase appears within minutes in the subarachnoid space, nasal cavity, nasal pharynx, and soft palate at the back of the throat (Figure 5; Leaston et al., 2021). Fluorescence quantum dots infused ICV again appear in the esophagus within minutes of injection suggesting unwanted waste from the brain may be cleared through a brain-to-gut pathway in addition to the meningeal and nasal lymphatics.

## Summary

Imaging awake animals using MRI will only improve as we move forward based on better acclimation protocols and restraining systems that eliminate all surgical preparations, ear bars, and other points of irritation. Better hardware and radiofrequency electronics will optimize space filling and SNR for complete brain coverage and the highest image quality. Image registration, data analysis and data interpretation will become automated assisted by artificial intelligence. All of these improvements should be standardized.

I have done my best to find and summarize all of the applications and publications on awake MRI in mice, rats, and marmoset monkeys. These animals are the most commonly used species for medical research, involving small bore high field MRI systems. While marmosets have the greatest translational value they have become very expensive limiting their access to most researchers. However, marmoset will play a significant role going forward in visual and social neuroscience and aging research. Indeed, marmosets are short-lived and offer many advantages over other non-human primates both in and out of the magnet (Tardif et al., 2011). The future will likely focus on rodents, and then more on rats than mice. Rats are phylogenetically closer to humans than mice (Gibbs et al., 2004) and are favored for neuroscience research (Iannaccone and Jacob, 2009; Ellenbroek and Youn, 2016) not to mention they have bigger brains which is better for MRI. PhMRI studies will continue to characterize new therapeutics not only for their ability to get into the brain and engage with their target but also for their potential liability in the areas of addiction and self-harm. In this area there should be more collaboration between pharmaceutical companies and academic labs. Other than epilepsy and Parkinson’s there are very few studies on awake functional MRI in animal models of neurological disorders. This can only improve as more transgenic models of neurodegenerative disease are generated in rats. The study of drug addiction is a key future area of research. The ability to expose a drug naïve brain to a known or potential addictive compound and record the acute and neuroadaptive effects to repeated exposer across multiple integrated neural circuits is an underdeveloped application. What are the functional changes in the brain that occur with drug addiction? Indeed, these types of prospective longitudinal studies on the same animal opens up new areas of research on changes in brain function in models of cancer, diabetes, cardiovascular disease, neurotrauma and the biggest disease of all – aging.

## Author Contributions

CF was the sole author and responsible for the content of this review.

## Conflict of Interest

The author has a financial interest in Animal Imaging Research, the company that makes the radiofrequency electronics and holders for awake animal imaging. The author also has a partnership interest in Ekam Solutions the company that develops 3D MRI atlases for animal research.

## Publisher’s Note

All claims expressed in this article are solely those of the authors and do not necessarily represent those of their affiliated organizations, or those of the publisher, the editors and the reviewers. Any product that may be evaluated in this article, or claim that may be made by its manufacturer, is not guaranteed or endorsed by the publisher.
